# To Dr. Adams

**Published:** 1809-03

**Authors:** John Jenkinson

**Affiliations:** Oxford


### To Dr. ADAMS.

Sir,

YOU would oblige me by informing me, either from your own extensive reading, or from inquiries amongst your medical friends, whether a complete abstinence from bodily exercise, and from the other means of throwing the lungs into injurious or unnecessary action, has ever been recommended in the treatment of Phthisis Pulmonalis; I mean, in the first stage of it? and, also whether it has ever been fairly put to the trial, and with what success? It has lately occurred to me, that a strict observance of the recumbent posture, combined with the same disuse of the powers of speech as is enjoined in hemorrhagies and wounds of the lungs, promises to be a most important addition to our other means of moderating the general circulation, and consequently the morbid action of the parts concerned in that fatal disease: but before I trouble you with any further account of the advantages to be reasonably expected from pursuing the plan, I should wish to know how far I have been anticipated in my ideas? The kind of rest I am speaking of, accords so well with the indications (I wish I could add of *cure*) as set down by the best authors, and combines so easily with the other remedies they have prescribed, (with the preservation of a moderate and equal temperature in the patient's apartments especially) that I confess I am rather sanguine with regard to the issue of any fair trials that may be made of them. The importance of every thing tending in the slightest degree to assist us in arresting the progress of phthisis pulmonalis requires no elucidation of mine, and therefore I shall content myself at present, with requesting the communication of whatever occurs to you upon the subject of this letter; and, *if necessary*, the publication of as much of it as is sufficient to procure me what assistance your Correspondents may be able or willing to afford me in the object of my inquiries.

I am, &c.

JOHN JENKINSON.

*Oxford, Feb.* 10, 1809.

